# Molecular Mechanisms of Nitric Oxide (NO) Signaling and Reactive Oxygen Species (ROS) Homeostasis during Abiotic Stresses in Plants

**DOI:** 10.3390/ijms22179656

**Published:** 2021-09-06

**Authors:** Kaiser Iqbal Wani, M. Naeem, Christian Danve M. Castroverde, Hazem M. Kalaji, Mohammed Albaqami, Tariq Aftab

**Affiliations:** 1 Department of Botany, Aligarh Muslim University, Aligarh 202 002, India; kaiseriqbal34@gmail.com (K.I.W.); naeemgaur@gmail.com (M.N.); 2 Department of Biology, Wilfrid Laurier University, Waterloo, ON N2L 3C5, Canada; dcastroverde@wlu.ca; 3 Department of Plant Physiology, Institute of Biology, Warsaw University of Life Sciences SGGW, Nowoursynowska 159, 02-776 Warsaw, Poland; hazem@kalaji.pl; 4 Institute of Technology and Life Sciences, National Research Institute, Falenty, Al. Hrabska 3, 05-090 Raszyn, Poland; 5 Department of Biology, Faculty of Applied Science, Umm Al-Qura University, Makkah 21955, Saudi Arabia; mmbaqami@uqu.edu.sa

**Keywords:** abiotic stress, nitric oxide (NO), drought stress, heavy metal stress, soil salinity, reactive oxygen species (ROS), plant stress

## Abstract

Abiotic stressors, such as drought, heavy metals, and high salinity, are causing huge crop losses worldwide. These abiotic stressors are expected to become more extreme, less predictable, and more widespread in the near future. With the rapidly growing human population and changing global climate conditions, it is critical to prevent global crop losses to meet the increasing demand for food and other crop products. The reactive gaseous signaling molecule nitric oxide (NO) is involved in numerous plant developmental processes as well as plant responses to various abiotic stresses through its interactions with various molecules. Together, these interactions lead to the homeostasis of reactive oxygen species (ROS), proline and glutathione biosynthesis, post-translational modifications such as S-nitrosylation, and modulation of gene and protein expression. Exogenous application of various NO donors positively mitigates the negative effects of various abiotic stressors. In view of the multidimensional role of this signaling molecule, research over the past decade has investigated its potential in alleviating the deleterious effects of various abiotic stressors, particularly in ROS homeostasis. In this review, we highlight the recent molecular and physiological advances that provide insights into the functional role of NO in mediating various abiotic stress responses in plants.

## 1. Introduction

Various abiotic stressors such as heavy metals, high salinity, high or low temperature, UV radiation, and drought pose serious threats to plants and are detrimental to agriculture and the ecosystem, resulting in major losses [1,2]. Due to their sessile nature, plants have to counteract these stressors by developing effective strategies during evolution to survive in these harsh conditions. Under such conditions, plants initiate various responses at molecular, physiological, and cellular levels [3,4]. A common and convergent plant response to abiotic stresses is the production of redox molecules, including reactive oxygen species (ROS) and reactive nitrogen species (RNS) [5].

ROS (e.g., singlet oxygen, hydrogen peroxide, superoxide anion, hydroxyl radical) and RNS (e.g., nitric oxide or NO) have important signaling functions in plants. These can be observed via the interaction of ROS and NO to form a range of RNS, apart from both being able to modify proteins involved in NO and ROS metabolism, signaling, and homeostasis [6]. However, the accumulation of ROS and RNS buildup may result in cellular damage and inactivation of important signaling molecules; therefore, proper regulation of ROS/RNS homeostasis forms an important feature of abiotic stress tolerance and resistance [3,7].

**Figure 1:**
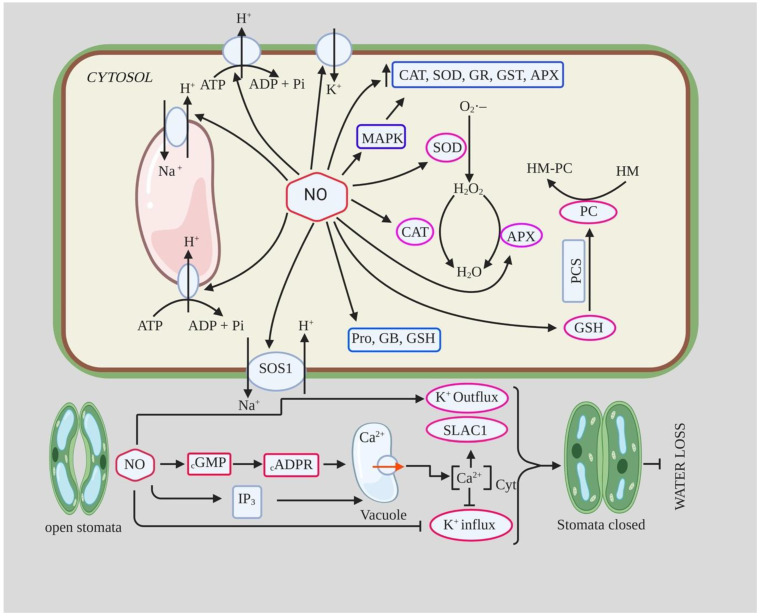
Schematic diagram showing the functional roles played by nitric oxide (NO) in plants exposed to drought, heavy metal, and salinity stress. NO enhances the activity of Na^+^/H^+^ antiporters on the vacuole membrane as well as the plasma membrane, such as SOS1, which helps in the removal of excess Na^+^ from cell cytoplasm. It also facilitates K^+^ ion entry into cells to maintain a balanced Na^+^/K^+^ ratio during salinity stress. NO up-regulates the activity of antioxidant enzymes, thiols, and compatible osmolytes, which protect the plants against salinity, drought, and HM stress by preventing membrane damage, ion and metal toxicity, osmotic stress, lipid peroxidation, and excess ROS production. It is involved in phytochelatin (PC) biosynthesis via GSH with the help of enzymes PCS; these PCs then help in sequestration of excess metals. NO also increases cytosolic free Ca^2+^ through cGMP and cADPR up-regulation. High cytosolic Ca^2+^ causes stomatal closure due to the activation of outward anion channels such as SLAC1 and inhibition of K^+^ inward channels. This helps in optimizing water usage during drought stress. Pro: Proline; GSH: Glutathione; GB: Glycine betaine; PCS: Phytochelatin synthase; HM-PC: Heavy metal-Phytochelatin complex; cADPR: Cyclic ADP-Ribose; cGMP: Cyclic GMP; CAT: Catalase; SOD: Superoxide dismutase; GR: Glutathione reductase; GST: Glutathione S-transferase; APX: Ascorbate peroxidase; IP_3_: Inositol triphosphate (it acts as a second messenger); Cyt: Cytosolic. Made with Biorender.com (Accessed on 3 September 2021).

**Figure 2:**
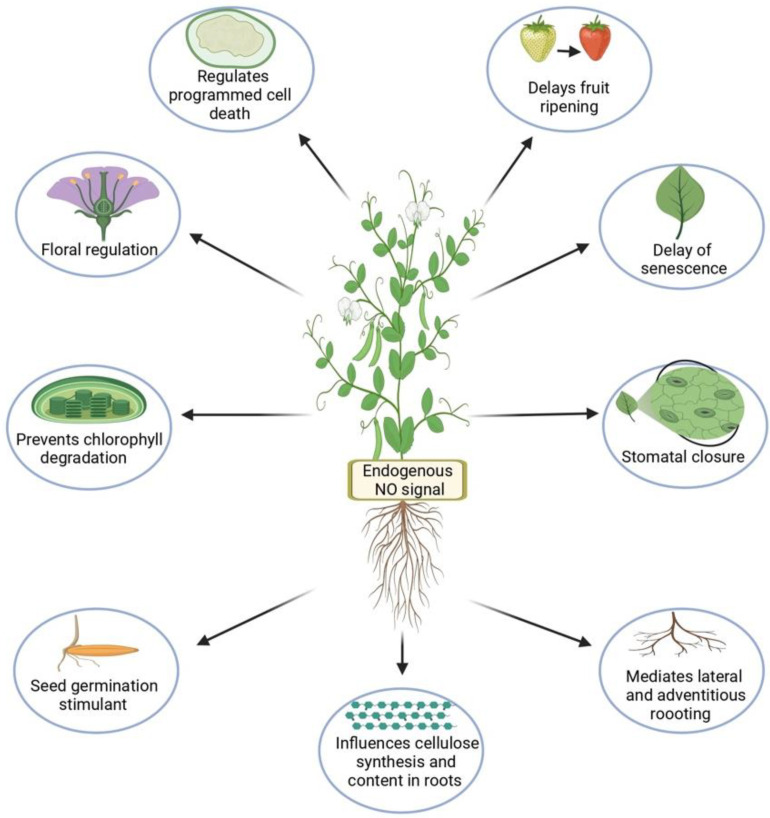
Role of No in plant growth and development. (Made with Biorender.com (accessed on 3 September 2021)).

Depending on their concentration, both NO and ROS act as double-edged swords. NO, an extremely reactive free radical and weak oxidant, is a unique type of diffusible signaling molecule first identified as an endothelium-derived relaxing factor [8]. Since then, it has been found to be involved in various physiological processes in mammals, such as neurotransmission, vasodilation, immune regulation, inhibition of platelet aggregation, apoptosis, and defense against microbes [9,10,11,12,13]. However, the emission of NO was first observed in plants by Klepper in *Glycine max* plants treated with herbicides, earlier than in animals [14]. NO is biologically active at 1 nmol/L concentration and participates in various signaling pathways to regulate plant growth and development [15]. In most of the experimental studies (Table 1 and Table 2), a 100 µM aqueous SNP (NO donor) solution has been shown as the best dose, with some studies showing 200 as well, releasing nanomolar amounts of NO. In plants, it is involved in the regulation of a number of physiological processes, such as stomatal movement (Figure 1) [16], photosynthesis [17], induction of apoptosis [18], senescence [19], floral regulation [20], seed germination [21], lateral root formation [22], adventitious root formation [23], regulation of cellulose content in roots (Figure 2) [24], and various responses to abiotic and biotic stresses, sometimes in interaction with other hormones [12,25,26]. It also plays an important role in regulating toxicity and levels of ROS (Figure 1), which is important for cytoprotection [27]. Because of its versatility and biological importance, it was rightly named “Molecule of the Year” by Science in 1992 [28].

ROS are produced as unwanted byproducts of various metabolic pathways in chloroplasts, mitochondria, peroxisomes, and legume nodules [52,53,54,55]. Their production is also induced by various abiotic and biotic stresses to regulate different processes, such as programmed cell death, pathogen defense, and stomatal behavior [5,56] as well as other signaling pathways that modulate plant functions [7]. When produced in excess and depending on the concentration and site of production, ROS can also oxidize and alter cellular components, modify proteins and lipids, and irreversibly damage DNA [57,58]. Therefore, plants require an appropriate homeostatic mechanism to maintain ROS levels within thresholds through various ROS interception systems to maximize their beneficial effects and minimize their cytotoxic effects on plants [54,59] (Figure 1).

## 2. Nitric Oxide (NO) Signaling under Abiotic Stresses

### 2.1. NO and Drought Stress

Drought is a major stress that limits plant growth and development and leads to reduced productivity worldwide. It leads to several interrelated physiological consequences that are harmful to plants by disrupting metabolism and damaging cells through oxidative stress [60]. One of the main consequences of drought stress is the gradual or rapid loss of water, leading to dehydration and cell death. Since water loss occurs primarily through stomata, this could be reduced by maintaining smaller stomatal openings. There is credible evidence that NO functions as a vital signaling molecule during both normal growth and development, and drought stress; exogenous application of NO mitigates the negative effects of drought, as seen in soybean, cucumber, and many other plants [30,61,62]. NO has been shown to mediate drought tolerance by activating the ROS scavenging enzyme system [63] and increasing osmolyte and proline metabolism [64]. NO also modulates water loss through abscisic acid-mediated stomatal response by acting as a secondary messenger in various signaling pathways, such as cyclic guanosine monophosphate (cGMP), mitogen-activated protein kinase (MAPK), and Ca^2+^ pathways [65]. During drought, NO also plays a significant role at the molecular level by regulating epigenetic changes and increased DNA demethylation, suggesting a possible role of NO in regulating the expression of key genes associated with drought, including antioxidant-acting genes and transcription factors [66,67,68].

Plants respond to drought stress through various adaptations, such as increased cuticle thickness due to deposition of wax crystals to reduce cuticular transpiration [69], development of an extensive root system to improve water supply, changes in leaf size, sunken stomata, and development of spongy tissue to reduce water loss [70]. These drought-tolerance mechanisms, generally classified as drought escape, drought avoidance, and drought recovery, usually involve NO and sometimes symbiotic rhizobacteria to modify the root system [22,71].

#### 2.1.1. NO and ROS-Mediated Oxidative Stress

During drought stress, plants produce excessive amounts of ROS (oxidative burst) due to a decrease in photosynthesis, leading to an excessive reduction in the electron transport chain and subsequent photooxidative stress [72]. Plants perform ROS detoxification through enzymatic mechanisms involving catalases (CATs), glutathione reductase (GR), glutathione S-transferase (GST), glutathione peroxidase (GPX), dehydroascorbate reductase (DHAR) monodehydroascorbate reductase (MDHAR), superoxide dismutase (SOD), peroxidase (POX), ascorbate peroxidase (APX), guaiacol peroxidase (GOPX), and metallothionein scavenging activity [73,74]. Non-enzymatic ROS detoxification mechanisms involve ascorbate (AsA), α-tocopherol, flavonoids, polyamines, glutathione (GSH), and carotenoids [73,74]. The antioxidant role of NO in protecting against cellular oxidative damage by reducing ROS is well documented [75]. Therefore, the NO-induced adaptive responses to cope with water deficit may be due to its direct action as an antioxidant, its role in stomatal closure, and its effects on root morphology [76] (Table 1).

NO mitigates the deleterious effects of ROS by limiting lipid peroxidation, increasing the rate of photosynthesis, and promoting antioxidants through various signaling pathways, e.g., the MAP kinase pathway [77,78]. Drought tolerance of plants is significantly enhanced by the activation of antioxidant enzyme systems such as CAT, SOD, GOPX, APX, DHAR, and GR [79]. The activity of CAT and SOD is down-regulated under drought, while application of NO up-regulates antioxidant activity as seen in hull-less barley [60]. The metalloenzyme SOD catalyzes the dismutation of superoxide to form H_2_O_2_, which is then converted to H_2_O and O_2_ by CAT and POX [80]. Activation of different isoforms of SOD under drought is considered as a strategy to counteract superoxide anion (O_2_^−^) accumulation in different cellular compartments [81,82]. SOD is quite important in preventing the reaction of O_2_^−^ with proteins, with unsaturated fatty acids for peroxidation, or with NO to form ONOO^−^; thus, transgenic plants overexpressing Cu or Zn isoforms of the SOD gene from *Puccinelia tenuiflora* have increased drought tolerance [83]. Fan et al. [66] showed that treatment with the NO donor, i.e., sodium nitroprusside (SNP), under drought, up-regulated the activities of SOD, CAT, and POX, resulting in lower ROS accumulation in such plants (Table 1). Increased malondialdehyde (MDA) content and electrolyte loss are important indicators of oxidative damage to cell membranes [84], and application of NO can counteract the negative effects of drought by reducing electrolyte loss and decreasing leaf H_2_O_2_ and MDA content [60]. NO generation has been reported to be up-regulated in *Cucumis sativus* seedlings upon polyamine (spermine and spermidine) treatment, and its exogenous application in the form of SNP counteracts lipid peroxidation and membrane damage induced by drought stress [8]. Moreover, they also found that exogenous NO application had no effect on endogenous polyamine levels in plants under drought stress but were positively correlated with mitigation of drought induced damage, indicating that polyamines act up-stream of NO in drought stress response.

#### 2.1.2. NO and Stomatal Closure during Drought

The exact mechanistic role of NO in ABA-mediated stomatal closure is not yet clear, but it has been proposed that NO is an important component of the ABA signaling pathway for stomatal closure [85]. NO acts downstream of the ABA signaling pathway and is an important component of the drought signaling network involved in the control of stomatal transpiration [30,61]. In a study conducted by Van Meeteren [86] on leaves of *Vicia faba* using SNP, NO gas, and ABA, they concluded that NO modifies stomatal opening through several pathways but is probably not critical for rapid ABA-induced stomatal closure. They found that NO/SNP application did not induce stomatal closure in epidermal cells, contradicting previous studies, whereas ABA application did induce stomatal closure [86]. In contrast, NO application on intact leaves resulted in a gradual decrease in stomatal conductance over a period of 1–2 h, indicating a possible interaction of a mesophyll-driven signal with NO in the induction of stomatal closure [86]. It has been proposed that stomatal movements are coordinated with carbon assimilation in the mesophyll through a complex interaction between sugars, malate, ion channels, and photosynthesis [87]. Furthermore, NO has been shown to increase cytosolic Ca^2+^ concentration in guard cells by up-regulating cGMP and cADPR, which act downstream of NO [88,89]. This increased Ca^2+^ concentration leads to activation of Cl channels (outward movement) and inactivation of K^+^ channels (inward movement), both of which result in stomatal closure [65,88]. Fan et al. [66] showed that exogenous SNP treatment in *Poncirus trifoliata* seedlings resulted in smaller stomatal openings and thus less water loss compared to the control. Investigating cellular mechanisms it was reported that NO in *Vicia faba* is involved in the reorganization of actin microfilaments under osmotic stress, which then regulate vacuolar dynamics to induce stomatal closure [90]. Moreover, De Sousa et al. [30] suggested that NO may also be involved in stomatal development and distribution between leaf sides under water stress conditions. In addition to stomatal closure, SNP treatment of plants under water stress caused an increase in leaf trichomes to maintain leaf water balance [91]. Overall, the application of NO prevents plant water loss to the atmosphere while maintaining plant productivity by altering morpho-anatomical and hydraulic properties [30]. Other than ABA, ethylene has also been found to be involved in NO-induced stomatal closure, with exogenous ethapon (ethylene releasing compound) application resulting in stomatal closure [65,92]. The ethylene-induced stomatal closure is associated with increased NO, Ca^2+^, and H_2_O_2_. The effect of ethylene on stomatal closure may be either direct due to modulation of NO level or indirect via modulation of ABA levels [65].

#### 2.1.3. NO and Drought-Responsive Genes

Various NO signaling partners and target proteins are involved in intracellular transduction cascades leading to gene activation or repression. Accordingly, NO has been reported to modulate transcript accumulation of various genes in several plant species [10,54]. NO can regulate protein function through posttranslational modifications, such as methylation [93,94], S-nitrosylation [95], and tyrosine nitration [96]. NO regulates the expression of several drought-sensitive genes, including antioxidant-responsive genes and transcription factors like *Glutathione transferase*, *OPR1* (*Oxophytodienoate reductase 1*), and *OPR2* (*Oxophytodienoate reductase 2*) [67,68]. In agreement, Feng et al. [97] suggested that overexpression of *HvAKT1* in barley enhances drought resistance due to efficient potassium ion uptake and regulation of NO and H_2_O_2_ signaling. Liu et al. [98] also revealed the role of *WD40- REPEAT 5a* (*WDR5a*) in drought stress tolerance, as this gene modulates the accumulation of NO in Arabidopsis by regulating nitric oxide synthase (NOS)-like activity. They further suggested that *WDR5a* increases the expression of stress-responsive genes, such as *KIN1*, *KIN2*, *RD22*, *RD29A*, and *RD29B*, by regulating NOS-like activity and NO accumulation in Arabidopsis. However, they found that NO synthesis mediated by nitrate reductase (NR) was not affected in *wdr5a* mutants, as *WDR5a* has been previously found to regulate yeast NOS-like activity, and they found similar levels of nitrite-induced NO (mediated by NR) in both wild and *wdr5a* mutants, with both showing similar sensitivity to NR inhibitor tungstate. Khan et al. [99] showed that NO induces the expression of the ABA biosynthetic gene *AtAO3*, especially under drought conditions; the regulation of ABA-related genes under drought suggests its involvement in drought responses. Moreover, they found that *atao3* and *atnced* ABA biosynthetic mutants showed less ABA and rapid wilting due to impaired stomatal closure under drought stress. These observations suggest a possible role of NO in ABA metabolism, with its involvement most required during drought.

Transcription factors are important molecular players that bind to gene promoters to activate or repress transcription. Interestingly, a number of transcription factors are NO-responsive and drought-dependent [70,100]. Overexpression of *SlWRKY8*, which belongs to the WRKY transcription factor superfamily, increases drought tolerance in tomato [101]. In contrast, *SlWRKY81* negatively regulates tomato drought tolerance by repressing NR activity, leading to reduced NO accumulation and eventually to reduced stomatal closure, which in turn increases water loss [102]. Silencing of *SlWRKY81* resulted in increased NO accumulation in guard cells due to increased NR expression, leading to more efficient stomatal closure and reduced water loss [102]. Thus, silencing of *SlWRKY81* can be used to increase tolerance in many drought-sensitive plants. Research on NO-mediated gene regulation in plants under drought stress is limited, and further research is needed to fully elucidate its role and paint a more comprehensive mechanistic picture.

### 2.2. NO and Metal/Metalloid Stress

Naturally occurring metallic elements with relatively higher atomic weight and density than water are called heavy metals (HMs) [103]. Contamination of the environment with HMs mostly occurs through anthropogenic activities, such as the use of metals and metal-containing compounds in agriculture and households, mining and smelting, and industrial production [20,104]. Several HMs are required for various physiological and biochemical functions as they are important for enzymatic functions; for example, Cu forms an important co-factor of several enzymes related to the attenuation of oxidative stress, such as CAT, POX, SOD, and cytochrome c oxidases [105,106]. However, excess metals cause damage to cells, tissues, and enzymes involved in metabolism and detoxification [107]. These HMs are translocated to various plant parts after root uptake and eventually enter the food chain [107]. Plant responses to these toxic HMs require a deeper and clearer understanding to develop HM-tolerant plants with phytoremediation potential [108].

One of the main consequences of HM stress in plants is the excessive ROS formation, due to Fenton and Haber–Weiss reactions and changes in the antioxidant system [109,110]. Certain metals such as lead and cadmium (Cd) are not directly involved in ROS formation, but they inhibit the antioxidant system and divert electrons from the electron transport chain, indirectly promoting ROS formation [111,112]. Both endogenous and exogenous NO may play a role in plant perception, signaling, and stress acclimation under HM stress [113]. NO is readily diffusible across membranes and is involved in the regulation of numerous physiological processes, including responses to HM stress [114].

The protective role of exogenous NO against HM stress has been confirmed by numerous studies. As reviewed by [113], the application of NO donors before or at the time of HMs treatment showed a positive correlation with chlorophyll content, biomass and root length. On the other hand, NO donor application correlated negatively with oxidative damage due to lipid peroxidation and ROS production (Table 2). However, HM tolerance by NO needs to be optimized because the application of a high NO concentration has cytotoxic properties [115].

#### 2.2.1. Cadmium Stress

Cadmium (Cd), a non-essential element and one of the most hazardous pollutants, can be toxic to animals even at non-phytotoxic concentrations [116]. It is rapidly taken up by plants due to its high mobility through Fe^2+^, Ca^2+^, Zn^2+^, and Mn^2+^ transporters, such as the ZIP IRT1 transporter [117]. As reviewed by Terrón-Camero et al. [114], NO donor application correlates negatively with HM uptake, except for Cd, which showed a positive correlation in about 40% of the studies. Cd accumulation in response to NO could most likely be due to stimulation of IRT1, which has been shown to be NO-dependent and inhibited in the presence of NO synthase inhibitor [118,119,120]. Sharing of IRT1 transporters under Cd stress leads to iron deficiency, which in turn results in NO-mediated up-regulation of FRO2 (Ferric reduction oxidase 2), IRT1 (Iron-regulated transporter 1), and FIT (FER-Like Fe deficiency induced transcription factor), leading to additional Cd accumulation [118]. Cd stress has been reported to induce endogenous NO generation, which reduces root growth due to shortening of the root elongation zone, an effect that is reversed by the NOS inhibitor L- NAME (N omega-Nitro-L-arginine methyl ester hydrochloride) [86]. However, exogenous NO may prevent the reduction of root growth in response to HM stress [86]. NO accumulation under Cd stress leads to the inhibition of root meristem in Arabidopsis due to the reduced AUX level in roots, and this inhibition was alleviated by the application of NO scavengers such as L- NAME and cPTIO (2-4-carboxyphenyl-4,4,5,5-tetramethylimidazoline-1-oxyl-3-oxide) [121]. These results suggest that NO has a negative effect on root growth and development under Cd stress. Moreover, the application of NO mitigates the negative effects of Cd stress on plant growth and development [44,122]. Khator et al. [123] found that NO confers increased Cd tolerance in *B. juncea* plants by maintaining cellular redox homeostasis and stimulating antioxidant production. NO acts as a potent ROS inhibitor under Cd stress by limiting lipid peroxidation [124]. Dong et al. [125] reported that the application of 250 µM SNP to peanut seedlings exposed to 200 µM Cd improved the antioxidant system, reduced MDA and O_2_^•−^ production, and increased growth parameters and chlorophyll content. Similarly, SNP treatment mitigated the negative effects of individual as well as combined Cd and lead stress on peppers by improving growth attributes and reducing H_2_O_2_ and MDA content [126]. NO has also been reported to regulate peroxisome proliferation, peroxule formation, and ROS-related metabolism in peroxisomes in response to Cd stress [50]. The inconsistent results on NO levels under Cd stress could arise due to the differences in the tissues studied, the type of species, the concentration used, and the duration of Cd stress (Table 2).

#### 2.2.2. Copper Stress

Although copper (Cu) is an essential micronutrient, its persistence in the environment is increasing due to agricultural (e.g., use of Cu-containing fungicides) and industrial activities and poses a threat to plants [127]. Although Cu is present in many subcellular compartments and is very important for plant metabolism by serving as an essential cofactor of various proteins such as SOD [128], it becomes phytotoxic at high concentrations, resulting in reduced plant biomass and mineral content, disruption of photosynthetic machinery, and increased oxidative stress [129].

Cu application has been reported to induce the formation of NO, which is mainly attributed to NOS, one of the most important enzymes in the production of NO [130]. However, Hu et al. [39] reported that early NO production in *Hordeum vulgare* under Cu stress was instead due to the activity of NR (not NOS), as the use of NR inhibitors resulted in decreased NO production in such plants. NO-mediated attenuation of Cu stress could be due to the up-regulation of defense-related genes or antioxidant enzyme activity [131]. In addition, NO also maintains the balance of cellular free metal concentrations by controlling their accumulation or excluding HMs in roots [131]. Application of SNP to *B. juncea* seeds increased their germination rate and alleviated Cu-induced oxidative stress due to enhancement of the antioxidant system, including SOD, GR, and APX, thereby lowering lipid peroxidation and H_2_O_2_ levels [132]. In a study by Yagci et al. [35], the application of NO (300 µM SNP) to Cu-stressed lettuce seedlings had a significant effect in attenuating the negative effects of Cu-induced genomic instability, DNA methylation, and retrotransposon polymorphism. SNP application also increased genome template stability (GTS) [133]. This protective role of exogenous NO for retrotransposon polymorphism and GTS under Cu stress might be due to NO enhancement of antioxidant enzyme activity (SOD, POX), preventing ROS accumulation and genome damage [133]. In *Chlamydomonas reinhardtii*, Cu stress-induced NO generation promotes proline synthesis, which helps to alleviate oxidative stress [134] (Table 2).

Several studies reported an early H_2_O_2_ burst after NO treatment [135]; however, H_2_O_2_ also functions as an important intracellular signaling molecule involved in plant adaptations to stress, including Cu toxicity. This suggests possible crosstalk between H_2_O_2_ and NO, which seems to be crucial for the protective effect of NO against Cu stress [135,136].

#### 2.2.3. Arsenic Stress

Arsenic (As) is a highly toxic metalloid whose toxicity causes various symptoms such as necrosis, decreased photosynthesis, and growth inhibition [112]. First, it causes an increase in ROS, leading to increased lipid peroxidation and protein carboxylation, which negatively affect metabolism and disrupt cellular ultrastructure [137]. Arsenic also inhibits the activity of enzymes by binding to their sulfhydryl groups (- SH), thereby hindering several important cellular functions [138]. Souri et al. [139] suggested that As tolerance of *Isatis capadoccica* (an As hyperaccumulator) may be related to NO as SNP treatment enhances plant growth under As stress, while the application of an NO scavenger and the NOS inhibitor L- NAME reduces plant growth. They concluded that SNP treatment correlated with increased proline, GSH, thiol, and antioxidant concentrations, such as CAT, APX, SOD, and GR, which prevent lipid peroxidation and H_2_O_2_ accumulation. GSH, an important thiol compound, is also involved in As tolerance by participating in the biosynthesis of phytochelatin (metal-binding peptide), which binds As III, preventing its toxicity [139]. This phytochelatin complex formation is regulated by NO and is considered to be one of the major mechanistic reasons for As hypertolerance in *I. capadoccica* [140]. The application of SNP to rice plants under As stress can increase primary root length and number of lateral roots compared to As alone, indicating the role of NO in mitigating the effects of As stress on root development [141].

It has also been reported that NO mediates adaptive responses to As stress via transcriptional modulation in *Oryza sativa* [142]. SNP treatment modulates the expression of several metal transporters such as natural resistance-associated macrophage protein (NRAMP), nodulin 26-like intrinsic protein (NIP), and ATP-binding cassette protein (ABC), stress-related genes (such as *GST*s and *CytP450*), transcription factors, and secondary metabolism genes [142,143]. These SNP-regulated genes are involved in As detoxification through vacuolar sequestration, reduction of As uptake, and efflux from the cell [142,143]. During As stress, NO mediates the reduction of ROS; its modulation of stress-related genes could be a strategy to cope with As stress by maintaining GSH biochemistry and redox homeostasis in cells [142,143]. Increased proline content in response to SNP treatment is most likely due to up-regulation of *P5CS1*, which is involved in proline biosynthesis; higher proline levels then prevent As uptake and enhance antioxidant potential [144,145]. As shown by Chandrakar et al. [146], both dimethylthiourea and NO provide As tolerance in soybean by up-regulating pyrroline-5-carboxylate synthetase, in parallel with the accumulation of sugars and proline, with NO being more efficient against As-induced oxidative stress (Table 2).

#### 2.2.4. Zinc Stress

Accumulation of zinc (Zn) in the environment occurs through both natural causes (volcanic eruptions, fires, and weathering) and anthropogenic activities (electroplating, mining, ore processing, ink and battery industries, and agrochemical application) [136,147]. Zn plays an important role in various redox reactions and is an essential cofactor of several enzymes, such as SOD, when present in the homeostatic range [148]. It is required by plants in trace amounts and is involved in several enzyme-catalyzed reactions; therefore, its toxicity impairs these reactions, which in turn can lead to oxidative stress, senescence, and retarded growth [149]. As reported by Kolbert et al. [150], Zn stress in Arabidopsis leads to reduced activities of CAT and APX and decreased GSH content, resulting in an overall excess of H_2_O_2_. The activity of S-nitrosoglutathione reductase is then inhibited, leading to an accumulation of NO-derived S-nitrosoglutathione (GSNO), which acts as a physiological source of NO and positive regulator of the *APX1* gene involved in ROS homeostasis [150]. These observations suggest that GSNO accumulation initiates NO signaling and cellular antioxidant machinery. Mechanistically, Abdel-Kader [151] proposed that NO-mediated Zn tolerance may be due to GSH and metal-binding proteins (metallothioneins) acting against high and low Zn levels, respectively. Zn-induced oxidative stress may also be alleviated by an enhanced antioxidant system for ROS detoxification and NO-mediated defense gene expression [152]. Under Zn stress, NO enhances the AsA-GSH cycle in plants by regulating GSH and AsA levels and related antioxidant enzymes such as GR and APX [49,153]. Exogenous NO has also been associated with reduced Zn uptake and translocation. Accordingly, the application of an NO donor to rice plants grown under Zn stress (2 μM) resulted in reduced Zn accumulation in roots and shoots compared to plants exposed to Zn alone [154]. Moreover, 100 μM SNP treatment on *Carthamus tinctorius* plants exposed to 500 mg kg^−1^ Zn resulted in reduced Zn accumulation, especially in shoots (Table 2).

#### 2.2.5. Other Heavy Metal Stresses (Lead, Chromium, Mercury)

Lead (Pb) is one of the most important environmental pollutants, especially in regions with high anthropogenic activities [155], and in toxic concentrations it negatively affects crop biomass as well as yield [156]. At high concentrations, it leads to reduced growth, ROS accumulation, irregular phytomorphology, and cell death [157]. As reported by Okant and Kaya [158], Pb stress leads to increased NO content in maize leaves, and this has also been reported for other plants in previous studies involving different HMs [159]. Interestingly, some of the studies showed that NO has a positive effect in mitigating the negative effects of Pb stress [121]. They further suggested that NO and H_2_O_2_ cooperate in triggering defense responses such as the increase of phenolic compound due to increased phenylalanine ammonia lyase activity and activation of antioxidant enzymes. Bai et al. [160] showed that exogenous application of NO (especially 100 μM SNP) on perennial rye grass under Pb stress enhanced the antioxidant enzyme system, reduced oxidative damage, and inhibited Pb translocation from roots to shoots.

Due to extensive industrial use, chromium (Cr) contamination has become a cause of environmental and scientific concern, with hexavalent Cr(VI) considered the most toxic among its various oxidation states [161]. Since it is not an essential element, there is no specific mechanism for its uptake and it competes with sulphur, phosphorus, and iron in carrier binding [162]. Once it enters the plants, it causes adverse effects in the plants from molecular level to whole plant level. Huang et al. [163] found that NO has the potential to attenuate Cr(VI) toxicity in tall fescue plants, improve the performance of the pigment system II, and improve overall physiological properties in these plants. NO has also been found to be helpful in mitigating Cr(VI)disadvantages in maize seedlings by suppressing lipoxygenase activity and enhancing antioxidant enzyme activities [164]. NO has been found to play a crucial role in germination and seedling development under Cr stress. Under Cr(VI)stress, the application of SNP improves seed germination and seedling development of tomato and increases the activity of protease and α-amylase hydrolyzing enzymes [165]. Furthermore, they reported an increase in nitrogen, proline, thiol content and antioxidants. These results suggest that exogenous NO application could be useful in Cr phytoremediation.

Mercury (Hg) is a non-essential element and contamination has become a major ecological problem due to the continuous release of Hg into ecological systems due to anthropogenic activities. Hg is introduced into agricultural soils through the use of Hg-containing compounds such as pesticides, fertilizers, lime, and soil amendments, resulting in Hg contamination [166]. Among the various forms of Hg, Hg^2+^ is the predominant and bioavailable form to plants [167]. The application of SNP showed positive correlation in attenuating the adverse effects of Hg toxicity in three soybean cultivars (Pusa-24, Pusa-37, and Pusa-40), where Pusa-37 showed better response to NO under Hg stress compared to the other cultivars [168]. They reported an increase in antioxidant response and the AsA-GSH cycle after SNP treatment in these plants. Chen et al. [169] reported that exogenous NO treatment attenuated Hg toxicity (in the form of HgCl_2_) in rice seedlings by preventing oxidative stress in leaves and promoting auxin transport in roots. They also reported a decrease in Hg absorption and transport and reduced ROS content.

Based on the above studies, we can conclude that NO is quite crucial in helping plants adapt under Pb, Cr, and Hg stress. However, research studies on the role of NO in regulating plant responses to Pb, Cr, and Hg stress are very limited compared to other metals. Therefore, further research is needed to explain the complex pathways and mechanisms involved in NO-mediated protection against these less-studied metals and other HMs.

### 2.3. NO and Salinity Stress

Soil salinity is assessed using the standard measurement ECe (electrical conductivity of a saturated soil solution). A soil sample with ECe equal to or greater than s 4 dS/m is classified as saline, which is comparable to 40 mM NaCl and produces an osmotic pressure of nearly 0.2 MPa [170]. Salinity stress affects plants in two ways: (1) First, high salinity in the soil impedes water uptake by plant roots, and (2) within the plant, high salinity affects metabolism and cell growth when it reaches toxic concentrations. Plants have evolved mechanisms of selection against NaCl in favor of other mineral nutrients that are normally present in soil at lower concentrations than NaCl. In general, plant roots take up water from the soil while excluding Na^+^ and Cl^−^ [171]. Plants growing in highly saline soils, i.e., halophytes (salt-tolerant plants), are able to perform this exclusion at higher salinities than glycophytes (salt-sensitive plants), with *Hordeum marinum* being able to perform this exclusion even at 450 mM NaCl [172].

The role of NO in salt tolerance has been studied in several plant species, and there is ample evidence that application of NO donor protects plants from salt stress by protecting against oxidative stress, maintaining ion homeostasis, regulating osmolyte accumulation, and improving physiological and biochemical parameters [173,174,175]. Treatment of pepper seedlings with 150 mM NaCl resulted in an increase in MDA and H_2_O_2_ content by ~100% and 87%, respectively, compared to the control [176]. However, they found that foliar application of 150 µM SNP to such seedlings decreased MDA and H_2_O_2_ content to 54% and 34%, respectively; it also improved leaf relative water content and antioxidant enzyme activity (SOD, POX, CAT) [176]. Ren et al. [175] reported that NO (10 µM SNP) pretreatment attenuated the inhibition of seed germination and early seedling growth of *Brassica chinensis* under salt stress. They found that SNP pretreatment increased antioxidant enzyme activity such as CAT, APX, and SOD and reduced H_2_O_2_ and MDA content, which reduced NaCl-induced oxidative damage. They also reported an increase in soluble sugar and proline content and increased K^+^/Na^+^ ratio in Radicula and Plumula. The maintenance of high K^+^/Na^+^ ratio and reduced Na+ accumulation is important for salt tolerance in plants as they reduce ion toxicity and contribute to the restoration of various metabolic processes [177]. The increased K^+^/Na^+^ ratio and decreased Na+ accumulation in NO-treated seedlings under salt stress is likely due to the inhibition of vacuolar Na+ compartmentation or Na^+^ influx through the plasma membrane of radicle [178]. Moreover, the increased K^+^ content and K^+^/Na^+^ ratio in NO-treated plants under salt stress could be due to decreased K+ efflux, an increase in competitive absorption sites, increased SOS1 transporter activity, and reduced H_2_O_2_ content [177,179]. In addition, NO was also able to induce the expression of H^+^-PPase and H^+^-ATPase, which detoxify the cell through Na^+^/H^+^ exchange, as well as the expression of AKT1-type K^+^ channels, ultimately leading to increased salinity tolerance [180]. NO assists sunflower seedlings to adapt to salinity stress (120 Mm NaCl) by regulating polyamine homeostasis by increasing the accumulation of polyamine biosynthetic enzymes, decreasing polyamine catabolism, and regulating their distribution [181]. Consistent with this, foliar application as well as pretreatment of NO also alleviates salinity-induced stress in broccoli plants by increasing antioxidant enzyme activity, decreasing MDA and H_2_O_2_ content, and improving glycine betaine, phenolics, and chlorophyll-*a* content [182]. NO acts as a cellular preservative that induces the expression of various genes controlling metabolic processes and also alters ROS content [12,183] (Table 3).

The AsA-glutathione cycle, which is regulated by S-nitrosylation and is an H_2_O_2_ detoxification pathway, plays an important role in providing salinity tolerance. The two major components of this cycle are MDHAR and APX, both of which have been reported to be induced under 150 Mm NaCl stress [188,189]. S-nitrosylation of APX at Cys32 increases its enzymatic activity, leading to reduction of H_2_O_2_ to water with AsA as substrate [133]. These observations establish a molecular link between ROS and NO signaling pathways. GSH and AsA act as redox buffers in addition to their radical-neutralizing function; therefore, up-regulation of the AsA-GSH cycle in NO treatment mitigates the deleterious effects of stress in plants [190]. In agreement with this, Kaya et al. [126] reported increased endogenous NO in salt-stressed pepper plants after salicylic acid treatment, resulting in up-regulation of the AsA-GSH cycle and various antioxidant enzymes.

## 3. Conclusions and Future Perspectives

During the last decade, studies have been conducted to decipher the functional role of NO in plant tolerance to abiotic stress, but further research is needed to fully elucidate the underlying mechanisms. NO can confer resistance to plants growing under various abiotic stresses such as drought, heavy metals, and salinity. Various NO donors have been used to understand its cytoprotective role; appropriate NO concentrations up-regulate the antioxidant system, maintain ROS homeostasis, and prevent oxidative damage (Figure 2).

As expected, NO content in plants increases during various abiotic stresses, as shown in most studies. Since most of these studies refer to ROS metabolism, other parameters somehow remain less characterized. These parameters, apart from ROS homeostasis, require more comprehensive analyses to clearly decipher their mechanisms of action and their complex interplay with other signaling components. Moreover, research should also focus on comparing the effects of different NO donors (in addition to SNP) in alleviating the negative effects of various abiotic stressors. Future studies could include the simultaneous use of multiple NO donors and/or the application of exogenous NO in gaseous form rather than in solution or suspension. Overall, these forward-looking and comprehensive experimental frameworks could help produce climate-resistant plants with high phytoremediation potential. These plants, grown in highly contaminated soils under a variety of stress conditions, will have far-reaching and long-term implications in addressing current agricultural and environmental challenges in a changing global climate.

## Figures and Tables

**Table 1:** Compilation of recent research studies investigating the role of NO in ameliorating drought stress in plants.

Plant Species	Drought Imposition	Concentration and Source of NO	Plant Response to NO	Reference
*Citrullus lanatus* (watermelon)	15% PEG 600	100 µM SNP	Reduction in MDA content Increased activity of APX Reduced oxidative damage Increased proline content	[29]
*Glycine max*	Withholding water	100 µM SNP	Reduced water loss and improved biomass due to alteration of stomatal characteristics and hydraulic conductivity	[30]
*Origanum majorana*	Withholding water	30 and 60 µM SNP	Improved water use efficiency Increased anthocyanin, soluble phenol, and flavonoid content Enhanced antioxidant capacity	[31]
*Brassica juncea*	10% PEG 6000	100 µM SNP	Antioxidant accumulation Reduction in MDA content Decreased ROS content	[32]
*Triticum aestivum*	15 and 30% PEG	0.5 mM SNP	Improved antioxidant defence Enhanced glyoxalase system resulting in restoration of leaf relative water content and proline content Enhanced endogenous NO production	[33]
*Zea mays*	Withholding water	50, 100, 150, and 200 µM SNP	100 µM SNP had a positive impact on chlorophyll content and water status Increased activity of CAT, SOD, and APX Improved activities of GR, GST, GOPX, nitrite and nitrate reductase activity	[34]

PEG: Polyethylene glycol; NO: Nitric oxide; SNP (Sodium nitroprusside); acts as NO donor; MDA: Malondialdehyde; APX: Ascorbate peroxidase; ROS: Reactive oxygen species; CAT: Catalase; SOD: Superoxide dismutase; GR: Glutathione reductase; GST: Glutathione S-transferase; GOPX: Guaiacol peroxidase.

**Table 2:** Compilation of recent studies on the effects of exogenous NO application on plants under heavy metal stress.

Table	Source and Concentration of Metal	Source and Concentration of Exogenous NO	Plant Species	Impact of NO Treatment	Reference
A: Copper	200, 400 µM CuSO_4_	200, 300 µM SNP	*Lactuca sativa*	Decreased DNA methylation Decreased genomic template instability Increased POX and SOD activity	[35]
	5, 25, 50 μM CuSO_4_	10 μM SNP	*Arabidopsis thaliana*	Increased cell viability	[36]
	200 µM CuCl_2_	100 μM SNP	*Lolium perenne*	Increased activity of SOD, CAT, APX and POX Increased chlorophyll content and photosynthesis Maintenance of Ion homeostasis	[37]
	0.2 mM Cu	0.05 mM SNP	*Nicotiana tabacum*	Increased chlorophyll content, RUBISCO activity and fresh weight	[38]
	450 µM CuSO4	200 µM SNP	*Hordeum vulgare*	Enhanced antioxidant enzyme activity and reduced lipid peroxidation Activation of AsA-GSH cycle	[39]
B: Cadmium	150 μM Cd	150 μM SNP	*Hordeum vulgare*	Decreased H_2_O_2_ and O_2_^−^ contents Increased AsA, and GSH content Increased expression of *HvAOX1a* gene	[40]
	200 μM CdSO_4_	200 μM SNP	*Catharanthus roseus*	Increased melatonin and endogenous NO concentration Increased activities of CAT, SOD, POX Decreased H_2_O_2_ and lipid peroxidation in roots	[41]
	100 μM CdSO_4_	50 μM SNP	*Oryza sativa*	Decreased Cd uptake by roots Restores RNS/ROS balance	[42]
	5, 7, or 9 μM CdCl_2_	300 μM SNP	*Vigna radiata*	Improvement adventitious formation in hypocotyl cuttings Prevents lipid peroxidation Enhanced antioxidant enzyme activity	[43]
	150 μM	100 μM SNP	*Solanum lycopersicum*	Reduced Cd uptake Enhanced AsA-GSH cycle Increased activities of SOD, CAT, GR, MDHAR and APX	[44]
C: Arsenic	75 mg/kg (NaAsO_2_)	100 μM SNP	*Brassica juncea*	Increased activities of antioxidant enzymes Increased thiol and proline biosynthesis Decreased As uptake	[45]
	50 μM (Sodium arsenate)	100 μM SNP	*Brassica* seedlings	Recovery of photosynthetic pigments Increased CAT and SOD activity resulting in decreased H_2_O_2_ and Recovery of AsA and GSH content	[46]
	150 μM (Sodium meta arsenite)	100 μM SNP	*Oryza sativa*	Enhanced nitrogen and thiol content Improved nitrate reductase and GOGAT activity Improved amino acid content	[47]
	1.5 mg L^−1^ As	0.1 mg L^−1^ SNP	*Pistia stratiotes * Leaves	Reduced ROS content Improved photochemical efficiency of PSII Maintained the integrity of cell organelles	[48]
D: Zinc	500 µM ZnSO_4_.7H_2_O	100 μM SNP	*Carthamus tinctorius*	Reduced Zn translocation from root to shoot Enhanced activity of AsA-GSH cycle and glyoxalase system enzymes	[49]
	100, 200 µM ZnO nanoparticles	100 μM SNP	*Triticum aestivum*	Decreased Zn accumulation in xylem and phloem saps Improved activity of AsA-GSH cycle	[50]
	0.05, 0.5 mM Zn (zinc sulfate) in nutrient solution	0.1 mM SNP	*Zea mays*	Increased chlorophyll content Decreased leaf and root Zn content Increased nitrogen and iron content	[51]

**Table 3:** Compilation of recent studies on the role of NO in ameliorating plant responses to salinity stress.

Experimental Plant	NaCl Concentration	Concentration and Source of NO	Impact of NO on Plants	Reference
*Jatropa curcas*	100 mM	75 μM SNP	Reduced oxidative damage Decreased toxic ion and ROS accumulation Increased accumulation of AsA and GSH Increased activity of CAT, SOD and GR	[183]
*Brassica oleracea* (Broccoli)	120 mM	0.02 mM SNP	Improved CAT, SOD, and POX activity Increased glycine betaine and total phenolic content Reduction in H_2_O_2_ and MDA content	[182]
*Crocus sativus* (Saffron)	50 and 100 mM NaCl	10 µM SNP	Improved growth Accumulation of compatible solutes Increased antioxidant enzyme activity and secondary metabolite biosynthesis	[184]
*Hylotelephium erythrostictum*	200 mM NaCl	50 μM SNP	Increased Na^+^ efflux and decreased K^+^ efflux Increased Ca^2+^ influx	[185]
*Brassica napus* (Rapeseed)	200 mM NaCl	10 μM SNP	Redox and ion homeostasis Modulation of antioxidant defence genes *SOS2* and *NHX1*	[186]
*Cicer arietinum* L. (chickpea)	50 and 100 mM NaCl	50 μM SNAP (*S*-nitroso-*N*-acetylpenicillamine)	Increased osmolyte accumulation Upregulation of CAT, SOD and APX genes Decreased electrolyte leakage, MDA and H_2_O_2_ content	[174]
*Gossypium* (Cotton) seedlings	100 mM NaCl	0.1 and 1.00 mM SNP	Increased K^+^ Decreased K^+^/Na^+^ ratio Increased antioxidant enzyme activity Decreased MDA content	[187]

SNP and SNAP are NO donors.

## Data Availability

Not applicable.
